# Observational study on the use of anti-inflammatory and IOP-lowering eye drops in patients with refractive regression after corneal laser surgery

**DOI:** 10.1097/MD.0000000000046138

**Published:** 2025-11-21

**Authors:** Mengman Gao, Zhenhong Fan, Xiujin Guo

**Affiliations:** a Department of Ophthalmology, The Second Hospital of Hebei Medical University, Shijiazhuang, China.

**Keywords:** corneal epithelial remodeling, corneal refractive surgery, refractive regression

## Abstract

This retrospective observational study describes the clinical outcomes and examines the association between refractive regression and corneal epithelial remodeling in patients treated with combined fluorometholone and intraocular pressure (IOP)-lowering eye drops after corneal laser surgery. Patients with refractive regression following corneal laser surgery who were treated with fluorometholone combined with either timolol maleate or brimonidine tartrate were included. Changes in uncorrected visual acuity (UCVA), spherical equivalent (SE), and central corneal epithelial thickness were recorded over time. Statistical analyses, including normality testing with the Shapiro–Wilk test and correlation analysis, were performed to evaluate the relationship between refractive outcomes and epithelial remodeling. Mean UCVA improved from 0.28 ± 0.10 to 0.08 ± 0.08 LogMAR, SE from − 1.49 ± 0.37 days to − 0.77 ± 0.29 days, and epithelial thickness from 68.22 ± 4.40 μm to 59.63 ± 4.99 μm (all *P* <.001). △ET was positively correlated with improvements in SE (*R* = 0.742, *P* <.001) and UCVA (*R* = 0.446). A linear relationship (y = 0.0559x + 0.1522) suggested that a 15 μm decrease in epithelial thickness corresponded to a 1 day improvement in SE. Patients with refractive regression after corneal laser surgery showed improvements in UCVA and SE, accompanied by reductions in central corneal epithelial thickness. These findings suggest that corneal epithelial remodeling may contribute to refractive outcomes in this setting. However, as this was an observational study without a control group, causality cannot be inferred. Further controlled studies are needed to validate these findings.

## 1. Introduction

Refractive regression is a relatively common complication following corneal laser refractive surgery such as LASIK and PRK, and it can negatively affect patients’ visual outcomes and satisfaction. The mechanisms underlying refractive regression are multifactorial, involving biomechanical, epithelial, and stromal remodeling, as well as postoperative inflammation.^[1]^ Among these, epithelial hyperplasia has been recognized as an important factor contributing to myopic regression.^[2]^ Previous studies using anterior segment optical coherence tomography have demonstrated that epithelial thickness can increase after refractive surgery and may be associated with a partial loss of the refractive effect.

Several strategies have been proposed to manage refractive regression. Surgical retreatment, such as LASIK enhancement or surface ablation, is often considered when residual stromal thickness allows. However, additional surgical procedures carry risks of complications,^[3]^ including corneal ectasia, haze, and infection. Non-surgical approaches have therefore attracted attention. Topical corticosteroids, which suppress postoperative inflammation and modulate wound healing, have been suggested as a means of reducing epithelial hyperplasia.^[4]^ In addition, the use of intraocular pressure (IOP)-lowering eye drops has been explored to counteract potential IOP elevations associated with corticosteroid treatment and to optimize corneal biomechanical stability. Despite their clinical use, data remain limited on the combined application of anti-inflammatory and IOP-lowering agents in patients with refractive regression.

The rationale for the present study arises from the lack of detailed investigations into how pharmacological management of refractive regression may relate to corneal epithelial remodeling and refractive outcomes. In particular, little is known about the temporal changes in epithelial thickness in patients undergoing such treatment and how these changes correlate with improvements in visual acuity and refractive error.

Therefore, this study aimed to investigate temporal changes in central corneal epithelial thickness in patients with refractive regression after corneal laser surgery and to examine their relationship with refractive outcomes. We also describe the clinical outcomes observed in patients treated with combined fluorometholone and IOP-lowering drops.

## 2. Materials and methods

### 2.1. Patients

This retrospective observational study included 18 patients (26 eyes) with refractive regression after corneal laser surgery, who were consecutively recruited from Department of Ophthalmology, The Second Hospital of Hebei Medical University (Shijiazhuang, China)between January 2023 to January 2024. The hospital is tertiary referral center serving patients from Shijiazhuhang and surrounding regions.

Inclusion criteria were: refractive regression defined as uncorrected visual acuity (UCVA) ≤ 20/20 and spherical equivalent (SE) ≥ −1.00 days after cycloplegic refraction, persisting more than 6 months following surgery; stable ocular condition without changes in axial length or lens status. Exclusion criteria were: history of corneal pathology or previous intraocular surgery; ocular surface disease such as severe dry eye or keratoconjunctivitis; systemic diseases known to affect the ocular surface; and incomplete clinical records.

All eligible patients during the study period were consecutively enrolled; no sampling was applied. As this was an exploratory study, the sample size was determined by the number of eligible cases available rather than by formal sample size calculation.

### 2.2. Research methods

#### 2.2.1. *Treatment and follow-up*

Patients were treated with topical fluorometholone (0.1%, 3–4 times daily) in combination with either timolol maleate (0.5%, twice daily) or brimonidine tartrate (0.2%, twice daily) for 1 month. The choice of IOP-lowering agent was determined according to patient characteristics: timolol maleate was prescribed as the first option, unless contraindicated (e.g., asthma, chronic obstructive pulmonary disease, bradycardia), in which case brimonidine tartrate was used. Physician judgment and patient history guided the selection. Patients were followed for a duration of 3 months.

#### 2.2.2. *Observation indicators*

Pre- and posttreatment evaluations included uncorrected visual acuity (UCVA), intraocular pressure (IOP), slit-lamp biomicroscopy, cycloplegic refraction, and central corneal epithelial thickness (within the 6-mm zone), measured using the Pentacam system. Mean values were used for statistical analysis. The change in epithelial thickness (△ET) was defined as the difference between epithelial thickness before and after treatment; The change in SE (△SE) was defined as the difference in SE following treatment. Correlations between △ET and changes in SE or UCVA were assessed.

In addition, the numbers of patients receiving timolol maleate and brimonidine tartrate were recorded. Due to the relatively small subgroup sizes, statistical comparisons between the 2 treatment groups were not meaningful and therefore were not performed.

#### 2.2.3. Statistical analysis

Data were analyzed using SPSS version 29.0 (IBM Corp., Armonk). Continuous variables were expressed as mean ± standard deviation. The normality of the data distribution was assessed using the Shapiro–Wilk test. For normally distributed variables, paired t-tests were applied for pre- and posttreatment comparisons; for non-normally distributed variables, the Wilcoxon signed-rank test was used. Correlation analyses between changes in corneal epithelial thickness, UCVA, and SE were performed using Pearson correlation for normally distributed variables and Spearman rank correlation for non-normally distributed variables. A *P*-value <.05 was considered statistically significant.

## 3. Results

### 3.1. Comparison of parameters before and after treatment

Before treatment, the mean uncorrected visual acuity (UCVA) was 0.28 ± 0.10 LogMAR, the mean SE was − 1.49 ± 0.37 days, and the average central corneal epithelial thickness was 68.22 ± 4.40 μm. Following 1 month of treatment, mean UCVA improved to 0.08 ± 0.08 LogMAR, SE improved to − 0.77 ± 0.29 days, and corneal epithelial thickness decreased to 59.63 ± 4.99 μm. All improvements were statistically significant (*P* <.001).

A total of 16 eyes (61.5%) received timolol maleate and 10 eyes (38.5%) received brimonidine tartrate in combination with fluorometholone. Comparison between the 2 treatment subgroups revealed no statistically significant differences in pretreatment or posttreatment UCVA, SE, or central corneal epithelial thickness (all *P* >.05). Therefore, subsequent analyses were performed for the entire cohort as a whole. (Table 1).

**Table 1:** Comparison of observation indexes before and after drug treatment.

Observation indicators	Before treatment	After treatment	*t*/z	*P*
Uncorrected visual acuity (LogMAR)	0.28 ± 0.10	0.08 ± 0.08	−3.941	<.001*
SE (D)	−1.49 ± 0.37	−0.77 ± 0.29	−3.946	<.001*
Corneal epithelial thickness (µm)	68.22 ± 4.40	59.63 ± 4.99	8.041	<.001*

SE = spherical equivalent.

* Statistical significance.

### 3.2. Correlation between △ET and △SE

**Figure 1.**
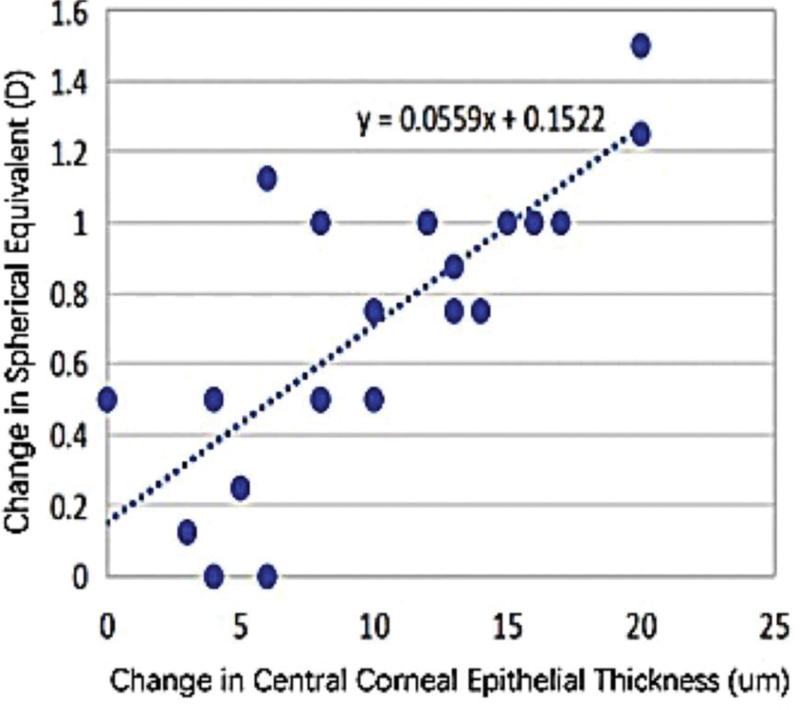
Changes in corneal epithelial thickness and SE in the central corneal region. SE = spherical equivalent.

The reduction in central corneal epithelial thickness (△ET) demonstrated a moderate positive correlation with improvement in UCVA (*R* = 0.446, *P* <.05). A strong positive correlation was also observed between △ET and the change in SE (△SE) (*R* = 0.742, *P* <.001), suggesting that more pronounced epithelial remodeling was associated with greater refractive improvement. According to the derived linear regression model (y = 0.0559x + 0.1522), a 15 μm reduction in epithelial thickness was estimated to correspond to approximately a 1.0 days shift in SE (Fig. 1).

## 4. Discussion

Corneal refractive surgery has become a widely adopted approach for the correction of refractive errors. However, refractive regression remains a prevalent postoperative complication that negatively impacts both visual quality and long-term patient satisfaction. Reports indicate that the incidence of refractive regression following LASIK in patients with high myopia can reach up to 40%.^[5]^ Current evidence suggests that several mechanisms may contribute to this phenomenon, including corneal epithelial and stromal remodeling, subepithelial haze, alterations in corneal curvature, and biomechanical instability.^[6]^

Our results demonstrated that after treatment, UCVA and SE both improved, accompanied by a reduction in central corneal epithelial thickness. These findings are consistent with previous studies indicating that epithelial hyperplasia plays an important role in postoperative refractive regression.^[7]^ Earlier studies using optical coherence tomography have reported that increases in central epithelial thickness of up to 18 μm have been observed within 1 day after surgery and that this thickening may result in mild undercorrection. Similarly, other studies^[8,9]^ have found that reductions in epithelial thickness after pharmacologic or surgical intervention may correspond to improvements in refractive outcomes.

The present study further supports the concept that epithelial remodeling is closely related to refractive outcomes in patients with regression after laser surgery. However, this study was not designed to evaluate the therapeutic effectiveness of any specific pharmacologic treatment, as there was no control or comparison group. Therefore, the results should be interpreted as descriptive observations rather than evidence of treatment efficacy.

Although fluorometholone and IOP-lowering agents are commonly used in the management of postoperative inflammation or elevated IOP, our study did not include a control group, and posttreatment IOP and adverse events were not recorded. As such, no conclusions regarding the safety profile or the comparative effects of these agents can be drawn from the current data. Treatment strategies for managing refractive regression include pharmacologic therapy and surgical enhancement procedures. Brimonidine tartrate has additional benefits, including pupil constriction, reduction of higher-order aberrations, and improvement in night vision.^[10]^ Corticosteroids inhibit corneal epithelial cell proliferation and migration, attenuate epithelial remodeling, and improve both refractive and visual outcomes.^[11]^ Cycloplegic agents such as compound tropicamide relax the ciliary muscle and reduce lenticular accommodation, contributing to refractive correction.^[12]^ Nonsteroidal anti-inflammatory drugs inhibit prostaglandin synthesis and thus reduce epithelial remodeling, helping to treat refractive regression.^[12,13]^ Dry eye has been suggested as a potential factor in postoperative regression in previous studies, although it was not evaluated in the present study.

In this study, a combination of fluorometholone and IOP-lowering drugs was employed to treat refractive regression. In the present study, posttreatment IOP and adverse events were not recorded; therefore, safety outcomes could not be assessed. This study has some limitations. First, the small sample size and single-center design may limit the generalizability of the findings. Second, the lack of a control group prevents causal inference regarding the treatment effect. Subgroup sizes for the timolol and brimonidine treatments were relatively small, precluding meaningful statistical comparison. Finally, posttreatment IOP data and adverse events were not collected, limiting assessment of safety. Future studies with larger, multicenter cohorts and prospective controlled designs are required to validate our findings.

In conclusion, our observational results suggest that reductions in corneal epithelial thickness are associated with improvements in refractive outcomes among patients experiencing regression after corneal laser surgery. These findings provide descriptive evidence supporting the role of epithelial remodeling in refractive stability and highlight the need for further prospective studies to clarify the potential contribution of pharmacologic therapy in this context.

## Author contributions

**Conceptualization:** Mengman Gao, Zhenhong Fan.

**Data curation:** Mengman Gao, Zhenhong Fan.

**Formal analysis:** Mengman Gao, Zhenhong Fan.

**Investigation:** Mengman Gao, Zhenhong Fan, Xiujin Guo.

**Methodology:** Mengman Gao, Xiujin Guo.

**Project administration:** Mengman Gao, Xiujin Guo.

**Supervision:** Xiujin Guo.

**Writing – original draft:** Mengman Gao, Zhenhong Fan.

**Writing – review & editing:** Mengman Gao, Zhenhong Fan, Xiujin Guo.
